# Systemic Lupus Erythematosus and Cardiovascular Disease: A Mendelian Randomization Study

**DOI:** 10.3389/fimmu.2022.908831

**Published:** 2022-06-06

**Authors:** Ning Gao, Minjian Kong, Xuebiao Li, Dongdong Wei, Xian Zhu, Ze Hong, Ming Ni, Yifan Wang, Aiqiang Dong

**Affiliations:** Department of Cardiovascular Surgery, The Second Affiliated Hospital of Zhejiang University School of Medicine, Hangzhou, China

**Keywords:** systemic lupus erythematosus, cardiovascular disease, Mendelian randomization, the causal link, genome-wide association study

## Abstract

**Background:**

Previous studies have shown that patients with systemic lupus erythematosus (SLE) tend to have a higher risk of cardiovascular disease (CVD), but the potential causal relationship between genetic susceptibility to SLE and CVD risk is not clear. This study systematically investigated the potential association between genetically determined SLE and the risk of CVD.

**Methods:**

The genetic tools were obtained from genome-wide association studies of SLE and CVD, with no overlap between their participating populations. Mendelian randomization (MR) analysis was performed using inverse variance weighting as the primary method. Simultaneously, a series of repeated analyses, sensitivity analyses, and instrumental variable strength evaluations were performed to verify the reliability of our results.

**Results:**

MR analysis showed that genetic susceptibility to SLE was associated with a higher risk of heart failure (OR=1.025, 95% CI [1.009-1.041], P=0.002), ischemic stroke (OR=1.020, 95% CI [1.005-1.034], P=0.009), and venous thromboembolism (OR=1.001, 95% CI [1.000-1.002], P=0.014). However, genetic susceptibility to SLE was negatively correlated with the risk of type 2 diabetes (OR=0.968, 95% CI [0.947-0.990], P=0.004). Sensitivity analysis found no evidence of horizontal pleiotropy or heterogeneity.

**Conclusion:**

Our MR study explored the causal role of SLE in the etiology of CVD, which would help improve our understanding of the basic disease mechanisms of SLE and provide comprehensive CVD assessment and treatment for SLE patients.

## Introduction

Cardiovascular disease (CVD) is defined as a group of cardiac and vascular diseases, including coronary artery disease (CAD), cerebrovascular disease, atrial fibrillation (AF), heart failure (HF), thrombotic disease, and heart metabolism-related diabetes. In 2020, CVD was responsible for nearly 19 million deaths worldwide, with an increase of 18.7% since 2010 (1). The mortality and prevalence of CVD vary widely according to the world’s regions, with the highest mortality rates in Eastern Europe and Central Asia, while those in North America and Western Europe were relatively low; North Africa and the Middle East had the highest CVD prevalence rates. CVD prevalence also varies among different populations: 11.5% among Caucasians, 10.0% among Blacks, 8.2% among Hispanics, 7.7% among Asians, and 14.6% among American Indians or Alaskan natives. CVD is one of the world’s leading causes of death and disability, accounting for 37% of deaths from non-communicable diseases in individuals under the age of 70 years (2). CVD etiology cannot be explained by any single cause and results from a combination of multiple outcomes (3). The occurrence and progression of CVD may be driven by the interactions between genetic and environmental factors and immune disorders (4).

Systemic lupus erythematosus (SLE) is a chronic autoimmune illness that frequently affects many organs and has a high prevalence and fatality rate (5). The first peak of death is mainly caused by SLE activity or complications, and the second peak is mainly caused by infection, CVD and so on (6). Several studies have reported that patients with SLE tend to have a higher prevalence of CVD (7). A cohort study of 252,676 patients with SLE and 758,034 controls in the United States showed that SLE was associated with a higher CAD risk (OR=1.42, 95% CI [1.40-1.44] (8). However, another observational study showed that in European populations, patients with SLE have a lower CAD risk (HR=0.61, 95% CI [0.48-0.77] (9). A cohort study showed that patients with SLE are at higher risk of developing IS compared to the general population (HR=2.2, 95% CI [1.7-2.8] (10). The risk of type 2 diabetes (T2DM) in patients with SLE remains controversial (11, 12). Case-control studies showed that SLE patients tend to have a higher risk of AF and HF than the general population (13). Notably, these observational studies may be limited by sample size and potential confounding factors. Factors such as side effects of SLE drugs and immune system disturbances may increase CVD risk. Therefore, the potential causal relationship between genetic susceptibility to SLE and CVD risk is unclear.

Confirmation of a causal association is challenging because of reverse causation and confounding between SLE and CVD risk. Mendelian randomization (MR) analysis is an emerging epidemiological research method that uses genetic variations as instrumental variables (IVs) to assess causal effects of exposure factors on outcomes (14). Due to the unique advantage of IVs, MR analysis is not affected by traditional confounding factors (15) and is in accordance with the normal causal order (16). Genome-wide association studies (GWAS) have provided robust and reliable IVs for MR studies. Therefore, we used MR analysis to explore whether there is a potential causal relationship between genetic susceptibility to SLE and CVD risk, apart from being mediated by other factors such as drug side effects.

## Methods

### Data Sources and Study Design

Summary-level statistical data for SLE were derived from a large meta-analysis of GWAS (17) including 7,219 cases and 15,991 controls. For the outcome dataset, GWAS data for HF were derived from FinnGen (https://www.finngen.fi/en) and included 23,397 cases and 19,4811 controls. The summary dataset for IS was obtained from the MEGASTROKE consortium and included 40,585 cases and 406,111 controls (18). Summary statistics for AF were derived from 5 cohort studies, including 60,620 cases and 970,216 controls (19). Single nucleotide polymorphisms (SNPs) for CAD were retrieved from a public GWAS meta-analysis, including 122,733 cases and 424,528 controls (20). Summary-level data for T2DM were derived from a GWAS that included 12,931 cases and 57,196 controls (21). The demographic profiles involved in this study were summarized in **Table 1**. The details of the GWAS are provided in **Supplementary Table 1**.

**Table 1:** Data sources and instrumental variables strength assessment.

Traits	Data sources	Sample size (cases/controls)	Ancestry	*R^2^(*%) for SLE (Total)	*F* for SLE (Total)
**Exposure**					
Systemic lupus erythematosus	Bentham et al	7,219/15,991	European		
**Outcomes**					
Heart failure	FinnGen	47,309/930,014	European	3.140	20.868
Venous thromboembolism	Neale lab (UK Biobank)	4,620/356,574	European	3.708	26.246
Ischemic stroke	MEGASTROKE	40,585/406,111	European	2.942	19.516
Atrial fibrillation	HUNT, UK Biobank, deCODE, DiscovEHR, MGI and AFGen	60,620/970,216	European	3.010	19.979
Coronary artery disease	CARDIoGRAMplusC4D and UK Biobank	122,733/424,528	European	3.126	21.993
Type 2 diabetes	GENEVA, WTCCC, FUSION, NuGENE and GERA	12,931/57,196	European	3.020	18.987

CARDIoGRAMplusC4D, Coronary Artery Disease Genome-wide Replication and Meta-analysis plus The Coronary Artery Disease Genetics; GENEVA, Gene Environment-Association Studies; WTCCC, Wellcome Trust Case Control Consortium; FUSION, Finland–United States Investigation of NIDDM Genetics; GERA, Resource for Genetic Epidemiology Research on Aging; NuGENE, Northwestern NuGENE project; HUNT, The Nord-Trøndelag Health Study; MGI, the Michigan Genomics Initiative; deCODE, the Collaborative Analysis of Diagnostic Criteria in Europe study; F=R^2^(N-K-1)/[K(1-R^2^)], R^2^= 2×(1-EAF)×EAF×(β/SD)^2^, SD=SE×N^1/2^, where EAF is the effect allele frequency, β is the estimated effect on adipokines, N is the sample size of the GWAS and SE is the standard error of the estimated effect.

**Figure 1:**
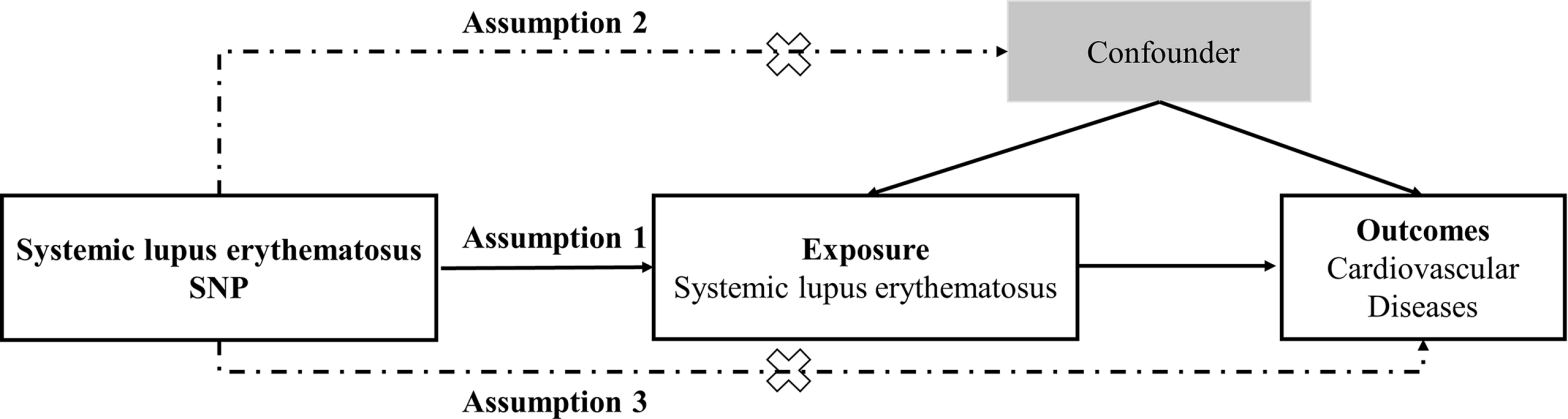
Study design flowchart of the Mendelian randomization study. The Mendelian randomization method is based on three hypotheses: 1. the instrumental variables is closely related to exposure; 2. instrumental variables is independent of any confounding factor; 3. instrumental variables affects the results only through exposure but not through other ways.

Two-sample MR study was conducted to evaluate the causal relationship between genetic susceptibility to SLE and CVD risk. SNPs were used as IVs (22). An overview of the research design is presented in **Figure 1**. The entire process satisfied the three main hypotheses of classical MR analysis: 1. IVs directly affected exposure; 2. IVs were not associated with confounders; and 3. IVs influenced the risk of outcomes directly through exposure, not through other pathways. All the original studies obtained ethical approval and informed consent. This study was conducted based on the latest (STROBE-MR) guidelines (23).

### Selection of IVs

All genetic variants significantly associated with SLE (*P* < 5 × 10^−8^) were considered as IVs. The corresponding linkage disequilibrium (LD) was tested to identify SNPs in a LD state. These SNPs were independent by pruning SNPs within a 10,000 kb window with an r^2^< 0.001 threshold. To exclude potential pleiotropic effects, we searched for secondary phenotypes of each SNP in PhenoScanner V2 (24). SNPs corresponding to the phenotype related to the outcomes were excluded, and the remaining SNPs were used for further analysis.

For the screened SNPs, we used variance (*R^2^*) and *F*-statistics to evaluate the strength of the IVs to avoid weak-tool bias (25, 26). The most recent and rigorous calculation method was adopted, *F*=*R* ^2^(*N*-*K*-1)/[*K*(1-*R* ^2^)], where *R^2^* refers to the cumulative explained variance of the selected SNP during exposure, *K* is the number of SNP for the final analysis, and *N* is the number of samples of the selected GWAS. If *F*>10, the correlation between the IVs and exposure was considered sufficiently strong, and the results of the MR analysis could avoid being affected by weak-tool bias.

### Statistical Analyses

We harmonized the aggregated SNP-SLE and SNP-CVD statistics to ensure that the alleles of each SNP were consistent between SLE and CVD. In the MR analysis, the inverse variance weighting (IVW) method of different models was used as the main analytical method according to heterogeneity (22). At the same time, median weighting (27), MR-Egger (28), Maximum-likelihood (29), MR-robust adjusted profile score (MR-RAPS) (30), and MR-pleiotropy residual sum and outlier (MR-PRESSO) (31) were used to infer the causal relationship. Each method makes different assumptions regarding the effectiveness of IVs. Median weighting is estimated when 50% of the IVs are invalid (27). Although the statistical ability of the MR-Egger method is low, it provides an estimate after correcting for multiple effects (28). MR-RAPS corrects horizontal multiplicity using robust adjusted contour scores, which reduces the deviation caused by the horizontal multiplicity (30). The MR-PRESO method can automatically detect outliers in IVW linear regression and remove outliers to provide corrected MR estimation (31). We used all these methods to explore causality comprehensively.

### Sensitivity Analyses

Various methods were introduced in this study for sensitivity analysis. First, Cochran’s Q test assessed the heterogeneity between individual SNP estimates and provided evidence for the selection of an appropriate analysis method. If the *p-*value was greater than 0.05, indicating no heterogeneity, the fixed-effects IVW method was considered as the main method; otherwise, the random-effects model was used. Second, we used the MR-Egger intercept method to test the horizontal pleiotropy of IVs (28). In the MR-Egger test, the intercept estimated the average horizontal pleiotropic effect across SNP, and if the *p*-value was less than 0.05, the IVW estimate might be biased. Third, we conducted a leave-one-out sensitivity test to examine whether a single SNP caused the results. Fourth, funnel and forest plots were generated to detect the existence of pleiotropy directly.

All statistical analyses were carried out using the **“**TwoSampleMR”, **“**MR-PRESSO”, and **“**mr.raps” packages in R software, Version 4.1.2. And all *p*-values were two-sided.

## Results

### Characteristics of the Selected SNPs and the CVD Outcomes

We extracted IVs that were significantly related to SLE from the GWAS (*P*< 5 × 10^−8^) and removed LD (*r^2^* <0.001,10,000-kb). Subsequently, SNPs related to CVD were retrieved from the PhenoScanner database. We excluded three SNPs, rs597808, rs6679677, and rs389884, associated with confounders (high blood pressure, diabetes, and coronary heart disease). We also deleted palindromic SNPs with a moderate allele frequency.

The screened SNPs were included in further analyses (**Supplementary Tables 2–7**). No evidence of weak-tool bias was found in the IVs strength test (*F*-statistic > 10) (**Table 1**).

### Causal Estimates of Genetic Susceptibility to SLE and CVD Risk

**Figure 2:**
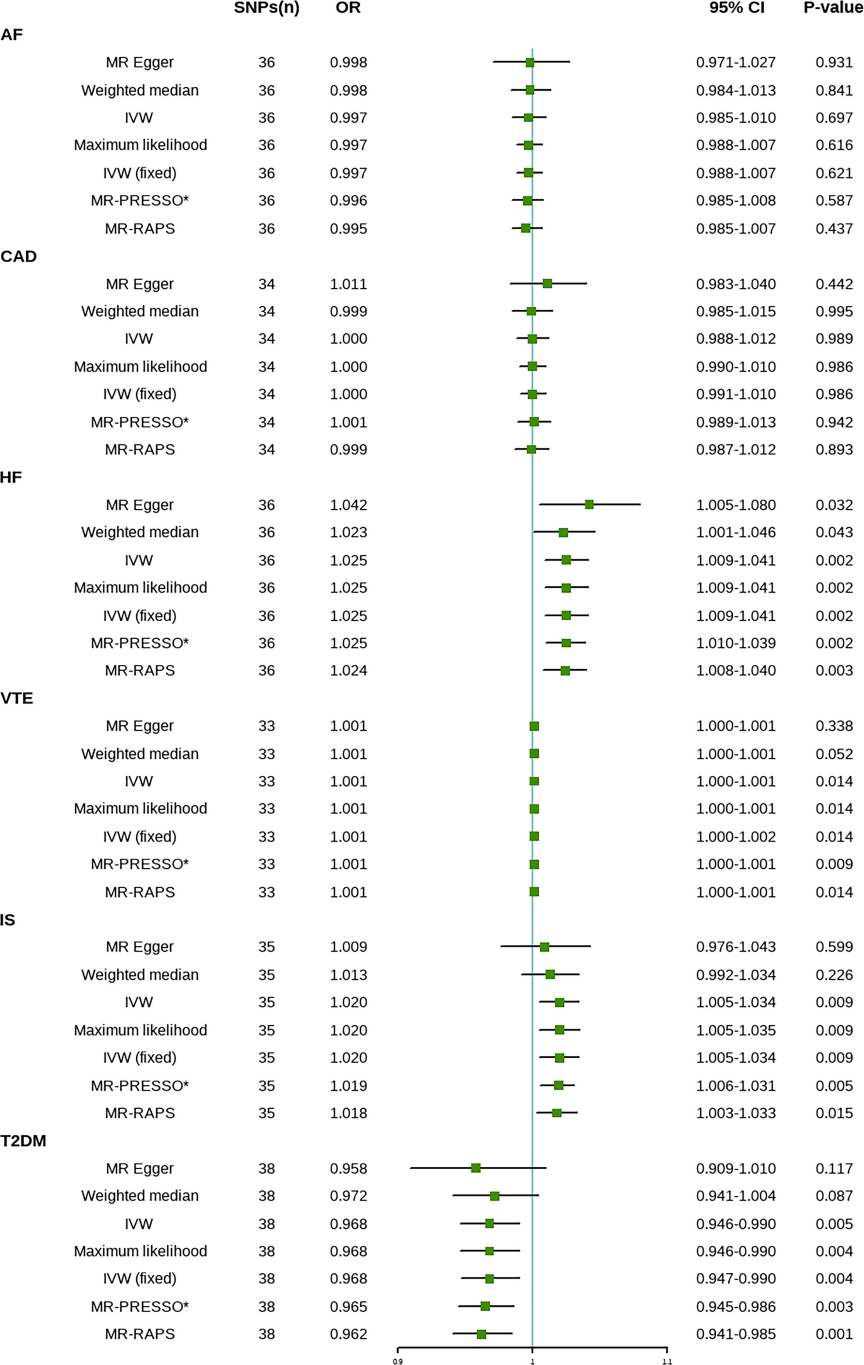
Mendelian randomization estimates of SLE on the risk for CVD. SNPs, Single nucleotide polymorphisms; OR, Odds ratio; CI, Confidence interval; IVW, inverse-variance weighted; IVW (fixed), fixed-effects inverse-variance weighted; MR-RAPS, MR-robust adjusted profile score; MR-PRESSO, MR-pleiotropy residual sum and outlier; *No outlier was detected; AF, atrial fibrillation; CAD, coronary artery disease; HF, heart failure; IS, ischemic stroke; T2DM, type 2 diabetes. VTE, Venous Thromboembolism.

The results are shown in **Figure 2**. The IVW method indicated that SLE is associated with a higher risk of HF, IS, and venous thromboembolism (VTE). Compared with the control group, the prevalence of HF in SLE patients had a 1.025-fold risk of HF (OR=1.025, 95% CI [1.009-1.041], *P*=0.002), a 1.020-fold risk of IS (OR=1.020, 95% CI [1.005-1.034], *P*=0.009), and a 1.001-fold risk of VTE (OR=1.001, 95% CI [1.000-1.002], *P*=0.014). A one-unit increase in the log-transformed OR of SLE reduced the risk of T2DM by 3.2% (OR=0.968, 95% CI [0.947-0.990], *P*=0.004). There was no significant difference in the prevalence of CAD (OR=1.000, 95% CI [0.991-1.010], *P*=0.986) and AF (OR=0.997, 95% CI [0.988-1.007], *P*=0.621) between SLE patients and controls (**Supplementary Figure 1**). The results of the maximum likelihood, MR-PRESSO, and MR-RAPS analyses were consistent with the IVW method. No outliers were identified using the MR-PRESSO method, indicating that the results are reliable. The risk calculation was based on the log OR of SLE, which may partly explain the low ORs.

### Sensitivity Analyses of MR

First, in the heterogeneity test, the *p*-values of Cochran’s Q statistics were all greater than 0.05, indicating no heterogeneity between SNPs (**Table 2**). Therefore, in this MR analysis, we used the fixed-effects IVW method as the main analytical method. Further, the MR-Egger regression intercept indicated limited evidence of pleiotropy in the IVs of SLE with any CVD. In addition, the leave-one-out method showed that the potential causal correlation between SLE and CVD risk was not driven by a single SNP (**Supplementary Figure 2**). Forest and funnel plots, which could more intuitively show heterogeneity, are shown in **Supplementary Figures 3, 4**.

**Table 2:** Pleiotropy and heterogeneity test of the SLE IVs from CVD GWAS.

Outcomes	Pleiotropy test			Heterogeneity test					
	MR-Egger			MR-Egger			Inverse-variance weighted		
	Intercept	SE	*p*	Q	Q_df	Q_*p*val	Q	Q_df	Q_*p*val
Heart failure	-0.006	0.006	0.329	32.948	34	0.519	33.930	35	0.520
Venous thromboembolism	1.83E-05	1.38E-04	0.895	17.646	31	0.974	17.663	32	0.981
Ischemic stroke	0.004	0.006	0.503	24.987	33	0.840	25.446	34	0.855
Atrial fibrillation	-4.23E-04	0.005	0.927	56.319	34	0.009	56.333	35	0.013
Coronary artery disease	-0.004	0.005	0.397	53.080	32	0.011	54.301	33	0.011
Type 2 diabetes	0.004	0.009	0.661	38.811	36	0.344	39.022	37	0.379

df, degree of freedom; MR, Mendelian randomization; Q, heterogeneity statistic Q.

## Discussion

We used MR for the first time to systematically explore potential causal effects between SLE susceptibility and CVD risk. The results of this study suggest that genetic liability to SLE is associated with an increased risk of HF, IS, VTE, and a lower T2DM risk. Limited MR evidence supports a potential causal relationship between genetic susceptibility to SLE and AF and CAD risk.

As a complex autoimmune illness, systemic lupus erythematosus can accumulate in any body organ. Cardiovascular complications of SLE cause a second peak in SLE mortality (32). Although the general mortality and prognosis of SLE have improved to some extent, cardiovascular mortality remains high (6, 33). Increasing evidence suggests that the effect of SLE on CVD is independent. A meta-analysis of 20 observational studies showed that SLE patients had an increased risk of stroke, HF, and peripheral vascular disease, consistent with our results (34). Another meta-analysis showed that patients with SLE had a two to three times higher risk of stroke than controls (35). A case-control study showed that the prevalence of T2DM and hyperlipidemia was significantly higher in patients with SLE (36). An observational study of 18,575 patients with SLE and 92,875 controls found that SLE patients had a higher risk of HF, stroke, and cardiac death (37). Similarly, several observational studies have shown that SLE patients have a higher risk of CVD (8, 38). However, some studies have yielded conflicting results. A prospective study found no significant increase in the risk of stroke in SLE patients compared to controls (39). Observational studies have shown no significant difference in cardiovascular parameters between SLE patients and controls with similar CVD risk (40). Another study showed no evidence of a significant correlation between T2DM risk in SLE patients and controls (34).

Our results are inconsistent with most previous studies in terms of the association between SLE and T2DM risk. There are several possible reasons for this discrepancy. First, it is controversial whether SLE is an independent risk factor for T2DM. A meta-analysis showed that previous assessments of diabetes risk in patients with SLE were mostly significantly heterogeneous (34). One study noted that compared with controls, SLE patients did not have a high index of insulin resistance (IR) and had normal glucose tolerance and beta cell function (41). There may even be higher fasting insulin levels and higher pancreatic beta-cell secretory function in patients with SLE (42). Conversely, some studies have reported increased IR and hyperglycemia in SLE patients (43). Second, almost all patients included in the previous study were on medications. As one of the main drugs, glucocorticoids may increase the risk of diabetes in SLE patients (44). Third, the onset of T2DM is triggered by genetic and environmental factors, and we evaluated the association between SLE and T2DM from a genetic perspective. In addition, the MR study considered lifetime effects rather than short-term effects, which might explain the differences between our findings and previous literature. Therefore, clinicians should exercise caution when patients with SLE present with higher fasting glucose levels or IR. Drug side effects should be taken seriously to avoid confusion with primary diabetes. Given the high mortality rate and poor prognosis of SLE and the inevitable side effects of drugs, glucose testing remains a necessity.

Owing to many interfering factors in traditional observational studies, the exact mechanism of the increased risk of CVD in patients with SLE remains controversial. Antiphospholipids and other autoantibodies, drugs such as glucocorticoids, hyperlipidemia, and systemic inflammation may increase CVD risk (45). Abnormal platelet activation often occurs in SLE patients, which may lead to the development and progression of CVD (46). Simultaneously, the disorder of fat factor levels in patients with SLE may also increase CVD risk (47). Complement activation and endothelial injury are common in SLE patients as one of the possible mechanisms of CVD development (48). Differences in drug use might be another confounding factor. As the main treatment, steroids and hydroxychloroquine (HCQ) often cause elevated blood sugar, obesity, and dyslipidemia, leading to bias in observational studies (49). Therefore, glucocorticoid use is an important explanation for the increased CVD risk in SLE patients (44). A cohort study demonstrated a five-fold increased risk of CVD in SLE patients using prednisolone (> 20 mg/day) across all age groups (50). The cardiovascular effects of HCQ, another essential drug, are controversial. The main reasons for this may be differences in treatment duration and drug combinations. Some studies have suggested that HCQ and immunosuppressants may increase CVD risk (51). Conversely, HCQ combined with low-dose aspirin prevents first-degree CVD in patients with SLE (52). Similarly, a retrospective cohort study showed that long-term HCQ treatment reduced the risk of CAD but not stroke (53), while another study showed that long-term HCQ use did not reduce cardiovascular events in patients with SLE (54). Combining multiple drugs to treat SLE is often common, making it more difficult to analyze the potential causal association between SLE and CVD risk.

Our study has several strengths. First, MR analysis of genetic susceptibility to other autoimmune diseases and CVD risk has recently been reported (55), but no MR studies have analyzed the potential causal association between SLE and CVD risk. Second, our genetic knowledge of SLE and CVD has been further expanded with large-scale GWAS meta-analyses. These large-scale GWAS have provided a more precise correlation. This MR analysis used the latest GWAS datasets of exposures and outcomes to comprehensively investigate the potential relationship between SLE and CVD, avoiding the traditional confounding factors and inverse causality. Third, we repeated the analysis using multiple methods and obtained consistent results. Sensitivity analysis and IVs strength assessment were used to verify that the results were not subject to bias.

However, our study has some limitations. First, although we used various methods to analyze multiplicity, potential multiplicity could not be completely excluded. Fortunately, multiple analytical methods yielded consistent results, and no evidence of horizontal pleiotropy or heterogeneity was found, confirming this study’s findings. Second, SLE prevalence and mortality vary based on ethnicity. All participants involved in this MR analysis were Europeans, making it more difficult to explain the potential causal association between SLE and CVD in other populations. Third, the OR value was relatively low and should be interpreted carefully.

## Conclusion

This study provided evidence for a potential causal relationship between SLE and an increased risk of IS, HF, VTE, and a decreased risk of T2DM. Our research will help improve our understanding of the basic disease mechanisms of SLE and provide comprehensive CVD assessment and treatment for SLE patients. We look forward to further research aimed at reducing CVD morbidity and mortality in patients with SLE. Considering the magnitude of the causal effect, the MR estimates in this study should be interpreted with caution.

## Data Availability Statement

The original contributions presented in the study are included in the article/**Supplementary Material**, further inquiries can be directed to the corresponding author.

## Author Contributions

NG and AD designed the study and drafted the article. DW and MK conducted data acquisition. NG, MK, DW, MN, ZH, XZ, YW, and AD performed data analysis and manuscript revision. All authors contributed to the article and approved the submitted version.

## Funding

This research was funded by Zhejiang Health Major Science and Technology Program, National Health Commission Scientific Research Fund (WKJ-ZJ-2121) and the National Natural Science Foundation of China (81800210).

## Conflict of Interest

The authors declare that the research was conducted in the absence of any commercial or financial relationships that could be construed as a potential conflict of interest.

## Publisher’s Note

All claims expressed in this article are solely those of the authors and do not necessarily represent those of their affiliated organizations, or those of the publisher, the editors and the reviewers. Any product that may be evaluated in this article, or claim that may be made by its manufacturer, is not guaranteed or endorsed by the publisher.
