# Supplementary material for: Systemic Lupus Erythematosus and Cardiovascular Disease: A Mendelian Randomization Study
Source: Front Immunol. 2022 Jun 6;13:908831. doi: 10.3389/fimmu.2022.908831 (PMC9207262; doi:10.3389/fimmu.2022.908831)
Supplement: Supplementary file 1 [file DataSheet_1.docx]

Supplementary Material

## SupplementaryTable

Supplementary Table 1 Characteristics of the genome wide association studies used in this study.

| **Exposure** | **Data source**  **(or PMID)** | **Inclusion Criteria for SNP** | **Imputation Quality Control**  **(r2/ INFO score)** | **Case Definition** | **Unit** | **Covariate adjustments** | **Imputation platfrom** |
| --- | --- | --- | --- | --- | --- | --- | --- |
| Systemic Lupus Erythematosus | PMID: 29848360 | MAF >1% | info score > 0.8 | American College for Rheumatology diagnosis criteria for Systemic Lupus Erythematosus | logodds | Study specific , Sex and Age as minimum | The 1000G Phase1 reference panels |
| **Outcomes** | **Data source (or PMID)** | **Inclusion Criteria for SNP** | **Imputation Quality Control**  **(r2/ INFO score)** | **Case Definition** | **Unit** | **Covariate adjustments** | **Imputation platfrom** |
| Heart failure | 31919418 | Study specific | Study specific INFO > 0.5 or 0.8 | Clinical Diagnosis of Heart Failure of any aetiology with no inclusion criteria based on LV ejection fraction | logodds | Sex, age (except for single-sex studies) and PCs for appropriate individual studies | 1KGP phase 1 or 3, Hapmap 2 NCBI build 36, Haplotype Reference Consortium, the Estonian WholeGenome Sequence reference, a reference sample based on 15,220 WGS of Icelandic individuals |
| Venous thromboembolism | UK Biobank  (Neale lab) | MAF >1% | INFO >0.3 for MAF >3% INFO >0.6 for MAF 13% INFO >0.8 for MAF 0.5-1% INFO >0.9 for MAF 0.1-0.5% | Defined as Venous thromboembolism based on clinical and imaging criteria | logodds | Sex and genotype array, and accounted for relatedness and stratification | UK10K haplotype and HRC reference panels |
| Ischemic stroke | 29531354 | MAF ≥1% | INFO ≥ 0.5 | Defined as ischemic stroke or intracerebral hemorrhage based on clinical and imaging criteria | logodds | Study specific , Sex and Age as minimum | 1KGP phase 1v3 or similar |
| Atrial fibrillation | 30061737 | Study  specific | r2 > 0.3 | DiscovEGR participants with at least one electronic health record problem list entry or at least two diagnosis code entries for two separate clinical encounters on separate calendar days for ICD-10 I48: atrial fibrillation and flutter. | logodds | Sex , age, age2, first four PCs of ancestry | Haplotype Reference Consortium |
| Coronary artery disease | 29212778 | MAF >0.5% | r2 >0.3 or INFO >0.4 | inclusive CAD diagnosis (e.g. myocardial infarction, acute coronary syndrome, chronic stable angina, or coronary stenosis >50%) | logodds | Study-specific covariates and over-dispersion | 1KGP phase 1v3 or similar |
| Type 2 diabetes | 29358691 | Study specific | info score ≥ 0.7 | FG >= 7 mmol/L or on drug treatment or HbA1c >= 6.5% (depends on study) | logodds | Sex, age, PCs for ancestry under an additive genetic model | UK10K haplotype and 1000G Phase1 reference panels |

MAF, minor allele frequency;

**Supplementary Table 2 Mendelian randomization analysis of Systemic lupus erythematosus and Atrial fibrillation.**

| **SNP** | **Chr** | **Position** | **A1** | **A2** | **EAF** | **Systemic lupus erythematosus** | | | **Atrial fibrillation** | | |
| --- | --- | --- | --- | --- | --- | --- | --- | --- | --- | --- | --- |
|  |  |  |  |  |  | **Beta** | **SE** | ***P-*val** | **Beta** | **SE** | ***P-*val** |
| rs10048743 | 2 | 213890232 | T | G | 0.826 | -0.231 | 0.041 | 2.04E-08 | -0.009 | 0.009 | 0.322 |
| rs10200680 | 2 | 223961877 | T | C | 0.144 | -0.248 | 0.042 | 4.96E-09 | -0.012 | 0.010 | 0.231 |
| rs1078324 | 5 | 149202268 | A | C | 0.058 | -0.713 | 0.078 | 7.11E-20 | 0.012 | 0.014 | 0.421 |
| rs10912578 | 1 | 173251856 | G | A | 0.668 | -0.247 | 0.031 | 1.65E-15 | -0.007 | 0.007 | 0.346 |
| rs1143679 | 16 | 31276811 | A | G | 0.112 | 0.582 | 0.040 | 5.03E-48 | 0.000 | 0.011 | 0.986 |
| rs12094036 | 1 | 183558174 | C | T | 0.077 | -0.329 | 0.058 | 1.37E-08 | 0.013 | 0.012 | 0.284 |
| rs12524498 | 6 | 31444187 | T | G | 0.031 | -0.673 | 0.121 | 2.48E-08 | -0.011 | 0.025 | 0.659 |
| rs13136219 | 4 | 102743687 | T | C | 0.355 | -0.174 | 0.028 | 3.50E-10 | 0.004 | 0.007 | 0.552 |
| rs13332649 | 16 | 85966683 | G | A | 0.250 | -0.315 | 0.038 | 5.43E-17 | 0.007 | 0.008 | 0.349 |
| rs143123127 | 17 | 38007190 | A | G | 0.049 | 0.470 | 0.084 | 2.23E-08 | -0.051 | 0.024 | 0.034 |
| rs1464446 | 3 | 146601295 | T | G | 0.196 | -0.329 | 0.040 | 2.79E-16 | 0.010 | 0.009 | 0.260 |
| rs150180633 | 6 | 31010047 | T | C | 0.017 | 0.928 | 0.069 | 2.66E-41 | -0.069 | 0.042 | 0.097 |
| rs17849501 | 1 | 183542323 | T | C | 0.052 | 0.811 | 0.050 | 1.81E-59 | 0.023 | 0.016 | 0.158 |
| rs2459611 | 2 | 191939187 | T | C | 0.876 | 0.261 | 0.045 | 7.62E-09 | 0.025 | 0.011 | 0.021 |
| rs2573219 | 2 | 233288667 | C | A | 0.091 | 0.588 | 0.043 | 1.13E-42 | -0.001 | 0.011 | 0.925 |
| rs268124 | 2 | 65654364 | T | C | 0.724 | 0.186 | 0.032 | 8.60E-09 | 0.002 | 0.008 | 0.826 |
| rs28361029 | 6 | 31220203 | A | G | 0.094 | -0.386 | 0.061 | 3.27E-10 | 0.023 | 0.026 | 0.362 |
| rs34703115 | 2 | 40282854 | C | T | 0.027 | -0.616 | 0.105 | 4.08E-09 | 0.009 | 0.021 | 0.667 |
| rs35000415 | 7 | 128585616 | T | C | 0.121 | 0.588 | 0.042 | 1.86E-45 | 0.006 | 0.010 | 0.591 |
| rs35251378 | 19 | 10459969 | A | G | 0.283 | -0.236 | 0.032 | 3.61E-13 | -0.004 | 0.008 | 0.643 |
| rs353608 | 11 | 35101738 | G | A | 0.511 | 0.186 | 0.028 | 2.93E-11 | 0.002 | 0.007 | 0.774 |
| rs3747093 | 22 | 21984379 | A | G | 0.207 | 0.262 | 0.035 | 2.88E-14 | 0.029 | 0.008 | 0.001 |
| rs4388254 | 5 | 133428601 | T | C | 0.042 | 0.378 | 0.060 | 3.71E-10 | -0.013 | 0.016 | 0.408 |
| rs4661543 | 1 | 15229101 | G | T | 0.854 | 0.274 | 0.042 | 9.40E-11 | -0.014 | 0.010 | 0.161 |
| rs4916215 | 1 | 173314540 | T | C | 0.715 | 0.223 | 0.034 | 5.07E-11 | -0.012 | 0.008 | 0.116 |
| rs58688157 | 11 | 625085 | G | A | 0.264 | -0.223 | 0.034 | 2.97E-11 | 0.004 | 0.008 | 0.561 |
| rs58721818 | 6 | 138243739 | T | C | 0.031 | 0.658 | 0.076 | 3.38E-18 | -0.015 | 0.020 | 0.466 |
| rs6671847 | 1 | 161478810 | A | G | 0.505 | 0.199 | 0.029 | 6.64E-12 | -0.021 | 0.007 | 0.002 |
| rs6889239 | 5 | 150457771 | C | T | 0.238 | 0.278 | 0.032 | 2.19E-18 | -0.002 | 0.008 | 0.775 |
| rs7097397 | 10 | 50025396 | A | G | 0.366 | -0.186 | 0.029 | 8.60E-11 | 0.006 | 0.007 | 0.387 |
| rs73050535 | 12 | 5012503 | T | C | 0.022 | -0.713 | 0.124 | 9.11E-09 | 0.012 | 0.027 | 0.653 |
| rs7768653 | 6 | 106574794 | T | C | 0.584 | -0.207 | 0.030 | 3.11E-12 | -0.012 | 0.007 | 0.085 |
| rs7823055 | 8 | 55511676 | T | G | 0.558 | -0.351 | 0.029 | 1.64E-34 | 0.006 | 0.007 | 0.392 |
| rs7899626 | 10 | 63825561 | T | C | 0.308 | 0.182 | 0.033 | 4.19E-08 | 0.002 | 0.007 | 0.828 |
| rs9274357 | 6 | 32632457 | T | C | 0.317 | 0.457 | 0.035 | 1.28E-38 | 0.004 | 0.024 | 0.875 |
| rs9852014 | 3 | 129084581 | G | A | 0.073 | 0.621 | 0.049 | 2.26E-36 | 0.012 | 0.013 | 0.336 |

Abbreviation: SNP, single nucleotide polymorphism; Chr, chromosome; EAF, effect allele frequency; SE, standard error;

Supplementary Table 3 Mendelian randomization analysis of Systemic lupus erythematosus and Coronary artery disease.

| **SNP** | **Chr** | **Position** | **A1** | **A2** | **EAF** | **Systemic lupus erythematosus** | | | **Coronary artery disease** | | |
| --- | --- | --- | --- | --- | --- | --- | --- | --- | --- | --- | --- |
|  |  |  |  |  |  | **Beta** | **SE** | ***P-*val** | **Beta** | **SE** | ***P-*val** |
| rs10048743 | 2 | 213890232 | T | G | 0.837 | -0.231 | 0.041 | 2.04E-08 | -0.001 | 0.010 | 0.910 |
| rs10200680 | 2 | 223961877 | T | C | 0.145 | -0.248 | 0.042 | 4.96E-09 | 0.019 | 0.010 | 0.057 |
| rs1078324 | 5 | 149202268 | A | C | 0.060 | -0.713 | 0.078 | 7.11E-20 | -0.009 | 0.015 | 0.530 |
| rs10912578 | 1 | 173251856 | G | A | 0.669 | -0.247 | 0.031 | 1.65E-15 | 0.000 | 0.007 | 0.990 |
| rs1143679 | 16 | 31276811 | A | G | 0.107 | 0.582 | 0.040 | 5.03E-48 | 0.000 | 0.012 | 0.980 |
| rs12094036 | 1 | 183558174 | C | T | 0.089 | -0.329 | 0.058 | 1.37E-08 | -0.002 | 0.012 | 0.850 |
| rs12524498 | 6 | 31444187 | T | G | 0.030 | -0.673 | 0.121 | 2.48E-08 | 0.025 | 0.024 | 0.300 |
| rs13136219 | 4 | 102743687 | T | C | 0.372 | -0.174 | 0.028 | 3.5E-10 | -0.011 | 0.007 | 0.120 |
| rs13332649 | 16 | 85966683 | G | A | 0.207 | -0.315 | 0.038 | 5.43E-17 | 0.002 | 0.008 | 0.810 |
| rs1464446 | 17 | 38007190 | A | G | 0.193 | -0.329 | 0.040 | 2.23E-08 | -0.024 | 0.009 | 0.007 |
| rs17849501 | 3 | 146601295 | T | G | 0.048 | 0.811 | 0.050 | 2.79E-16 | 0.033 | 0.016 | 0.041 |
| rs2431697 | 6 | 31010047 | T | C | 0.427 | -0.223 | 0.029 | 2.66E-41 | 0.009 | 0.007 | 0.190 |
| rs2459611 | 1 | 183542323 | T | C | 0.887 | 0.261 | 0.045 | 1.81E-59 | 0.009 | 0.012 | 0.430 |
| rs2573219 | 2 | 191939187 | T | C | 0.102 | 0.588 | 0.043 | 7.62E-09 | 0.005 | 0.012 | 0.690 |
| rs268124 | 2 | 233288667 | C | A | 0.725 | 0.186 | 0.032 | 1.13E-42 | 0.016 | 0.008 | 0.047 |
| rs34703115 | 2 | 65654364 | T | C | 0.033 | -0.616 | 0.105 | 8.6E-09 | 0.006 | 0.020 | 0.750 |
| rs35000415 | 6 | 31220203 | A | G | 0.114 | 0.588 | 0.042 | 3.27E-10 | 0.000 | 0.011 | 0.970 |
| rs35251378 | 2 | 40282854 | C | T | 0.287 | -0.236 | 0.032 | 4.08E-09 | -0.013 | 0.008 | 0.084 |
| rs353608 | 7 | 128585616 | T | C | 0.546 | 0.186 | 0.028 | 1.86E-45 | -0.008 | 0.007 | 0.240 |
| rs3747093 | 19 | 10459969 | A | G | 0.227 | 0.262 | 0.035 | 3.61E-13 | -0.009 | 0.009 | 0.330 |
| rs4274624 | 11 | 35101738 | G | A | 0.765 | -0.560 | 0.033 | 2.93E-11 | -0.014 | 0.008 | 0.100 |
| rs4388254 | 22 | 21984379 | A | G | 0.062 | 0.378 | 0.060 | 2.88E-14 | -0.011 | 0.017 | 0.520 |
| rs4661543 | 5 | 133428601 | T | C | 0.866 | 0.274 | 0.042 | 3.71E-10 | -0.015 | 0.010 | 0.140 |
| rs4916215 | 1 | 15229101 | G | T | 0.732 | 0.223 | 0.034 | 9.4E-11 | 0.003 | 0.008 | 0.720 |
| rs58688157 | 1 | 173314540 | T | C | 0.271 | -0.223 | 0.034 | 5.07E-11 | 0.014 | 0.008 | 0.074 |
| rs58721818 | 11 | 625085 | G | A | 0.031 | 0.658 | 0.076 | 2.97E-11 | -0.017 | 0.020 | 0.400 |
| rs6671847 | 6 | 138243739 | T | C | 0.484 | 0.199 | 0.029 | 3.38E-18 | -0.006 | 0.007 | 0.390 |
| rs6889239 | 1 | 161478810 | A | G | 0.276 | 0.278 | 0.032 | 6.64E-12 | -0.001 | 0.008 | 0.920 |
| rs7097397 | 5 | 150457771 | C | T | 0.369 | -0.186 | 0.029 | 2.19E-18 | -0.005 | 0.007 | 0.510 |
| rs73050535 | 10 | 50025396 | A | G | 0.021 | -0.713 | 0.124 | 8.6E-11 | 0.030 | 0.023 | 0.190 |
| rs73068668 | 12 | 5012503 | T | C | 0.073 | -0.315 | 0.057 | 9.11E-09 | 0.023 | 0.014 | 0.091 |
| rs7768653 | 6 | 106574794 | T | C | 0.610 | -0.207 | 0.030 | 3.11E-12 | -0.006 | 0.007 | 0.440 |
| rs7823055 | 8 | 55511676 | T | G | 0.559 | -0.351 | 0.029 | 1.64E-34 | 0.012 | 0.007 | 0.096 |
| rs7899626 | 10 | 63825561 | T | C | 0.312 | 0.182 | 0.033 | 4.19E-08 | -0.017 | 0.008 | 0.027 |

Supplementary Table 4 Mendelian randomization analysis of Systemic lupus erythematosus and Heart failure.

| **SNP** | **Chr** | **Position** | **A1** | **A2** | **EAF** | **Systemic lupus erythematosus** | | | **Heart failure** | | |
| --- | --- | --- | --- | --- | --- | --- | --- | --- | --- | --- | --- |
|  |  |  |  |  |  | **Beta** | **SE** | ***P-*val** | **Beta** | **SE** | ***P-*val** |
| rs10048743 | 2 | 213890232 | T | G | 0.817 | -0.231 | 0.041 | 2.04E-08 | -0.005 | 0.014 | 0.723 |
| rs10200680 | 2 | 223961877 | T | C | 0.169 | -0.248 | 0.042 | 4.96E-09 | -0.019 | 0.015 | 0.202 |
| rs1078324 | 5 | 149202268 | A | C | 0.042 | -0.713 | 0.078 | 7.11E-20 | -0.024 | 0.028 | 0.384 |
| rs10912578 | 1 | 173251856 | G | A | 0.684 | -0.247 | 0.031 | 1.65E-15 | -0.015 | 0.012 | 0.215 |
| rs1143679 | 16 | 31276811 | A | G | 0.117 | 0.582 | 0.040 | 5.03E-48 | 0.007 | 0.017 | 0.696 |
| rs12094036 | 1 | 183558174 | C | T | 0.092 | -0.329 | 0.058 | 1.37E-08 | -0.027 | 0.019 | 0.162 |
| rs12524498 | 6 | 31444187 | T | G | 0.020 | -0.673 | 0.121 | 2.48E-08 | -0.064 | 0.040 | 0.108 |
| rs13136219 | 4 | 102743687 | T | C | 0.367 | -0.174 | 0.028 | 3.50E-10 | 0.017 | 0.012 | 0.149 |
| rs13332649 | 16 | 85966683 | G | A | 0.277 | -0.315 | 0.038 | 5.43E-17 | -0.003 | 0.013 | 0.787 |
| rs1464446 | 3 | 146601295 | T | G | 0.177 | -0.329 | 0.040 | 2.79E-16 | -0.023 | 0.015 | 0.110 |
| rs150180633 | 6 | 31010047 | T | C | 0.004 | 0.928 | 0.069 | 2.66E-41 | -0.045 | 0.089 | 0.615 |
| rs17849501 | 1 | 183542323 | T | C | 0.038 | 0.811 | 0.050 | 1.81E-59 | 0.006 | 0.029 | 0.848 |
| rs2431697 | 5 | 159879978 | C | T | 0.388 | -0.223 | 0.029 | 2.60E-14 | 0.001 | 0.011 | 0.951 |
| rs2459611 | 2 | 191939187 | T | C | 0.947 | 0.261 | 0.045 | 7.62E-09 | 0.011 | 0.025 | 0.670 |
| rs2573219 | 2 | 233288667 | C | A | 0.085 | 0.588 | 0.043 | 1.13E-42 | 0.000 | 0.020 | 1.000 |
| rs268124 | 2 | 65654364 | T | C | 0.625 | 0.186 | 0.032 | 8.60E-09 | 0.006 | 0.012 | 0.621 |
| rs28361029 | 6 | 31220203 | A | G | 0.049 | -0.386 | 0.061 | 3.27E-10 | 0.028 | 0.026 | 0.286 |
| rs34703115 | 2 | 40282854 | C | T | 0.034 | -0.616 | 0.105 | 4.08E-09 | -0.092 | 0.032 | 0.004 |
| rs35000415 | 7 | 128585616 | T | C | 0.150 | 0.588 | 0.042 | 1.86E-45 | 0.024 | 0.016 | 0.117 |
| rs35251378 | 19 | 10459969 | A | G | 0.261 | -0.236 | 0.032 | 3.61E-13 | -0.009 | 0.013 | 0.476 |
| rs353608 | 11 | 35101738 | G | A | 0.534 | 0.186 | 0.028 | 2.93E-11 | 0.007 | 0.011 | 0.521 |
| rs3747093 | 22 | 21984379 | A | G | 0.313 | 0.262 | 0.035 | 2.88E-14 | 0.019 | 0.012 | 0.121 |
| rs4274624 | 2 | 191958656 | T | C | 0.769 | -0.560 | 0.033 | 9.73E-66 | -0.025 | 0.013 | 0.063 |
| rs4388254 | 5 | 133428601 | T | C | 0.113 | 0.378 | 0.060 | 3.71E-10 | 0.009 | 0.018 | 0.607 |
| rs4661543 | 1 | 15229101 | G | T | 0.933 | 0.274 | 0.042 | 9.40E-11 | -0.003 | 0.023 | 0.913 |
| rs4916215 | 1 | 173314540 | T | C | 0.816 | 0.223 | 0.034 | 5.07E-11 | -0.010 | 0.014 | 0.489 |
| rs58688157 | 11 | 625085 | G | A | 0.206 | -0.223 | 0.034 | 2.97E-11 | -0.002 | 0.014 | 0.868 |
| rs58721818 | 6 | 138243739 | T | C | 0.018 | 0.658 | 0.076 | 3.38E-18 | 0.007 | 0.042 | 0.873 |
| rs6671847 | 1 | 161478810 | A | G | 0.454 | 0.199 | 0.029 | 6.64E-12 | 0.001 | 0.011 | 0.952 |
| rs6889239 | 5 | 150457771 | C | T | 0.286 | 0.278 | 0.032 | 2.19E-18 | -0.016 | 0.012 | 0.188 |
| rs7097397 | 10 | 50025396 | A | G | 0.370 | -0.186 | 0.029 | 8.60E-11 | -0.031 | 0.012 | 0.008 |
| rs73050535 | 12 | 5012503 | T | C | 0.005 | -0.713 | 0.124 | 9.11E-09 | -0.023 | 0.080 | 0.780 |
| rs73068668 | 19 | 55763262 | A | G | 0.083 | -0.315 | 0.057 | 4.40E-08 | 0.001 | 0.020 | 0.961 |
| rs7768653 | 6 | 106574794 | T | C | 0.533 | -0.207 | 0.030 | 3.11E-12 | 0.003 | 0.011 | 0.810 |
| rs7823055 | 8 | 55511676 | T | G | 0.569 | -0.351 | 0.029 | 1.64E-34 | 0.000 | 0.011 | 0.974 |
| rs7899626 | 10 | 63825561 | T | C | 0.392 | 0.182 | 0.033 | 4.19E-08 | -0.013 | 0.012 | 0.266 |

Supplementary Table 5 Mendelian randomization analysis of Systemic lupus erythematosus and Venous thromboembolism.

| **SNP** | **Chr** | **Position** | **A1** | **A2** | **EAF** | **Systemic lupus erythematosus** | | | **Venous thromboembolism** | | |
| --- | --- | --- | --- | --- | --- | --- | --- | --- | --- | --- | --- |
|  |  |  |  |  |  | **Beta** | **SE** | ***P-*val** | **Beta** | **SE** | ***P-*val** |
| rs10048743 | 2 | 213890232 | T | G | 0.860 | -0.231 | 0.041 | 2.04E-08 | 1.21E-05 | 3.83E-04 | 0.975 |
| rs10200680 | 2 | 223961877 | T | C | 0.147 | -0.248 | 0.042 | 4.96E-09 | 5.03E-04 | 3.74E-04 | 0.179 |
| rs1078324 | 5 | 149202268 | A | C | 0.056 | -0.713 | 0.078 | 7.11E-20 | 8.97E-05 | 5.74E-04 | 0.876 |
| rs10912578 | 1 | 173251856 | G | A | 0.674 | -0.247 | 0.031 | 1.65E-15 | -2.93E-04 | 2.85E-04 | 0.304 |
| rs1143679 | 16 | 31276811 | A | G | 0.101 | 0.582 | 0.040 | 5.03E-48 | 8.67E-04 | 4.44E-04 | 0.051 |
| rs12094036 | 1 | 183558174 | C | T | 0.080 | -0.329 | 0.058 | 1.37E-08 | 1.01E-05 | 4.88E-04 | 0.983 |
| rs13019891 | 2 | 113829869 | T | G | 0.459 | -0.562 | 0.029 | 1.65E-83 | -2.90E-04 | 2.67E-04 | 0.278 |
| rs13136219 | 4 | 102743687 | T | C | 0.375 | -0.174 | 0.028 | 3.50E-10 | -3.85E-04 | 2.74E-04 | 0.159 |
| rs13332649 | 16 | 85966683 | G | A | 0.226 | -0.315 | 0.038 | 5.43E-17 | -6.86E-04 | 3.16E-04 | 0.030 |
| rs143123127 | 17 | 38007190 | A | G | 0.035 | 0.470 | 0.084 | 2.23E-08 | -2.90E-04 | 7.25E-04 | 0.689 |
| rs1464446 | 3 | 146601295 | T | G | 0.193 | -0.329 | 0.040 | 2.79E-16 | 7.00E-05 | 3.36E-04 | 0.835 |
| rs150180633 | 6 | 31010047 | T | C | 0.013 | 0.928 | 0.069 | 2.66E-41 | -4.78E-04 | 1.17E-03 | 0.682 |
| rs17849501 | 1 | 183542323 | T | C | 0.052 | 0.811 | 0.050 | 1.81E-59 | 5.56E-04 | 5.94E-04 | 0.349 |
| rs2431697 | 5 | 159879978 | C | T | 0.433 | -0.223 | 0.029 | 2.60E-14 | -1.67E-04 | 2.67E-04 | 0.531 |
| rs2459611 | 2 | 191939187 | T | C | 0.903 | 0.261 | 0.045 | 7.62E-09 | 2.84E-04 | 4.55E-04 | 0.532 |
| rs268124 | 2 | 65654364 | T | C | 0.733 | 0.186 | 0.032 | 8.60E-09 | 1.90E-04 | 2.99E-04 | 0.525 |
| rs34703115 | 2 | 40282854 | C | T | 0.025 | -0.616 | 0.105 | 4.08E-09 | -5.27E-04 | 8.48E-04 | 0.535 |
| rs35000415 | 7 | 128585616 | T | C | 0.112 | 0.588 | 0.042 | 1.86E-45 | 4.16E-04 | 4.20E-04 | 0.322 |
| rs35251378 | 19 | 10459969 | A | G | 0.294 | -0.236 | 0.032 | 3.61E-13 | -2.32E-04 | 2.91E-04 | 0.425 |
| rs353608 | 11 | 35101738 | G | A | 0.509 | 0.186 | 0.028 | 2.93E-11 | -3.20E-05 | 2.65E-04 | 0.904 |
| rs3747093 | 22 | 21984379 | A | G | 0.195 | 0.262 | 0.035 | 2.88E-14 | 3.36E-04 | 3.38E-04 | 0.320 |
| rs4274624 | 2 | 191958656 | T | C | 0.775 | -0.560 | 0.033 | 9.73E-66 | -1.12E-05 | 3.17E-04 | 0.972 |
| rs4388254 | 5 | 133428601 | T | C | 0.036 | 0.378 | 0.060 | 3.71E-10 | -4.97E-04 | 7.06E-04 | 0.481 |
| rs4916215 | 1 | 173314540 | T | C | 0.729 | 0.223 | 0.034 | 5.07E-11 | 1.16E-04 | 2.98E-04 | 0.696 |
| rs58688157 | 11 | 625085 | G | A | 0.280 | -0.223 | 0.034 | 2.97E-11 | 7.79E-05 | 2.95E-04 | 0.792 |
| rs58721818 | 6 | 138243739 | T | C | 0.032 | 0.658 | 0.076 | 3.38E-18 | 3.19E-04 | 7.53E-04 | 0.672 |
| rs6889239 | 5 | 150457771 | C | T | 0.235 | 0.278 | 0.032 | 2.19E-18 | -9.59E-06 | 3.12E-04 | 0.975 |
| rs7097397 | 10 | 50025396 | A | G | 0.366 | -0.186 | 0.029 | 8.60E-11 | -1.60E-04 | 2.74E-04 | 0.559 |
| rs73050535 | 12 | 5012503 | T | C | 0.024 | -0.713 | 0.124 | 9.11E-09 | -2.58E-04 | 8.63E-04 | 0.765 |
| rs73068668 | 19 | 55763262 | A | G | 0.076 | -0.315 | 0.057 | 4.40E-08 | -1.23E-04 | 5.04E-04 | 0.808 |
| rs7768653 | 6 | 106574794 | T | C | 0.599 | -0.207 | 0.030 | 3.11E-12 | 3.36E-04 | 2.70E-04 | 0.213 |
| rs7823055 | 8 | 55511676 | T | G | 0.568 | -0.351 | 0.029 | 1.64E-34 | -1.34E-04 | 2.69E-04 | 0.618 |
| rs7899626 | 10 | 63825561 | T | C | 0.300 | 0.182 | 0.033 | 4.19E-08 | 2.01E-04 | 2.92E-04 | 0.491 |

Supplementary Table 6 Mendelian randomization analysis of Systemic lupus erythematosus and Ischemic stroke.

| **SNP** | **Chr** | **Position** | **A1** | **A2** | **EAF** | **Systemic lupus erythematosus** | | | **Ischemic stroke** | | |
| --- | --- | --- | --- | --- | --- | --- | --- | --- | --- | --- | --- |
|  |  |  |  |  |  | **Beta** | **SE** | ***P-*val** | **Beta** | **SE** | ***P-*val** |
| rs10048743 | 2 | 213890232 | T | G | 0.849 | -0.231 | 0.041 | 2.04E-08 | -0.007 | 0.014 | 0.605 |
| rs10200680 | 2 | 223961877 | T | C | 0.142 | -0.248 | 0.042 | 4.96E-09 | 0.017 | 0.015 | 0.243 |
| rs1078324 | 5 | 149202268 | A | C | 0.055 | -0.713 | 0.078 | 7.11E-20 | -0.007 | 0.023 | 0.775 |
| rs10912578 | 1 | 173251856 | G | A | 0.688 | -0.247 | 0.031 | 1.65E-15 | -0.017 | 0.011 | 0.123 |
| rs1143679 | 16 | 31276811 | A | G | 0.118 | 0.582 | 0.040 | 5.03E-48 | 0.001 | 0.017 | 0.975 |
| rs12094036 | 1 | 183558174 | C | T | 0.077 | -0.329 | 0.058 | 1.37E-08 | -0.021 | 0.019 | 0.270 |
| rs12524498 | 6 | 31444187 | T | G | 0.021 | -0.673 | 0.121 | 2.48E-08 | 0.013 | 0.052 | 0.806 |
| rs13136219 | 4 | 102743687 | T | C | 0.364 | -0.174 | 0.028 | 3.50E-10 | -0.002 | 0.010 | 0.873 |
| rs13332649 | 16 | 85966683 | G | A | 0.240 | -0.315 | 0.038 | 5.43E-17 | -0.001 | 0.012 | 0.960 |
| rs143123127 | 17 | 38007190 | A | G | 0.035 | 0.470 | 0.084 | 2.23E-08 | -0.012 | 0.032 | 0.715 |
| rs1464446 | 3 | 146601295 | T | G | 0.190 | -0.329 | 0.040 | 2.79E-16 | 0.010 | 0.013 | 0.431 |
| rs150180633 | 6 | 31010047 | T | C | 0.041 | 0.928 | 0.069 | 2.66E-41 | 0.011 | 0.043 | 0.797 |
| rs17849501 | 1 | 183542323 | T | C | 0.052 | 0.811 | 0.050 | 1.81E-59 | 0.021 | 0.027 | 0.440 |
| rs2459611 | 2 | 191939187 | T | C | 0.893 | 0.261 | 0.045 | 7.62E-09 | -0.004 | 0.016 | 0.795 |
| rs2573219 | 2 | 233288667 | C | A | 0.094 | 0.588 | 0.043 | 1.13E-42 | 0.002 | 0.017 | 0.913 |
| rs268124 | 2 | 65654364 | T | C | 0.735 | 0.186 | 0.032 | 8.60E-09 | 0.010 | 0.011 | 0.390 |
| rs28361029 | 6 | 31220203 | A | G | 0.072 | -0.386 | 0.061 | 3.27E-10 | 0.018 | 0.028 | 0.523 |
| rs34703115 | 2 | 40282854 | C | T | 0.032 | -0.616 | 0.105 | 4.08E-09 | -0.058 | 0.036 | 0.103 |
| rs35000415 | 7 | 128585616 | T | C | 0.119 | 0.588 | 0.042 | 1.86E-45 | 0.026 | 0.015 | 0.088 |
| rs35251378 | 19 | 10459969 | A | G | 0.279 | -0.236 | 0.032 | 3.61E-13 | -0.022 | 0.011 | 0.048 |
| rs353608 | 11 | 35101738 | G | A | 0.521 | 0.186 | 0.028 | 2.93E-11 | 0.003 | 0.010 | 0.734 |
| rs3747093 | 22 | 21984379 | A | G | 0.212 | 0.262 | 0.035 | 2.88E-14 | 0.026 | 0.013 | 0.037 |
| rs4388254 | 5 | 133428601 | T | C | 0.052 | 0.378 | 0.060 | 3.71E-10 | 0.005 | 0.024 | 0.832 |
| rs4661543 | 1 | 15229101 | G | T | 0.869 | 0.274 | 0.042 | 9.40E-11 | 0.020 | 0.015 | 0.194 |
| rs4916215 | 1 | 173314540 | T | C | 0.735 | 0.223 | 0.034 | 5.07E-11 | -0.005 | 0.011 | 0.671 |
| rs58688157 | 11 | 625085 | G | A | 0.259 | -0.223 | 0.034 | 2.97E-11 | 0.008 | 0.012 | 0.467 |
| rs58721818 | 6 | 138243739 | T | C | 0.029 | 0.658 | 0.076 | 3.38E-18 | -0.017 | 0.032 | 0.585 |
| rs6889239 | 5 | 150457771 | C | T | 0.248 | 0.278 | 0.032 | 2.19E-18 | -0.001 | 0.011 | 0.967 |
| rs7097397 | 10 | 50025396 | A | G | 0.373 | -0.186 | 0.029 | 8.60E-11 | -0.013 | 0.010 | 0.196 |
| rs73050535 | 12 | 5012503 | T | C | 0.022 | -0.713 | 0.124 | 9.11E-09 | 0.005 | 0.046 | 0.921 |
| rs7768653 | 6 | 106574794 | T | C | 0.593 | -0.207 | 0.030 | 3.11E-12 | -0.004 | 0.010 | 0.681 |
| rs7823055 | 8 | 55511676 | T | G | 0.568 | -0.351 | 0.029 | 1.64E-34 | -0.007 | 0.011 | 0.516 |
| rs7899626 | 10 | 63825561 | T | C | 0.313 | 0.182 | 0.033 | 4.19E-08 | 0.013 | 0.011 | 0.238 |
| rs9274357 | 6 | 32632457 | T | C | 0.200 | 0.457 | 0.035 | 1.28E-38 | -0.024 | 0.025 | 0.343 |
| rs9852014 | 3 | 129084581 | G | A | 0.074 | 0.621 | 0.049 | 2.26E-36 | 0.034 | 0.020 | 0.086 |

Supplementary Table 7 Mendelian randomization analysis of Systemic lupus erythematosus and Type 2 diabetes.

| **SNP** | **Chr** | **Position** | **A1** | **A2** | **EAF** | **Systemic lupus erythematosus** | | | **Type 2 diabetes** | | |
| --- | --- | --- | --- | --- | --- | --- | --- | --- | --- | --- | --- |
|  |  |  |  |  |  | **Beta** | **SE** | ***P-*val** | **Beta** | **SE** | ***P-*val** |
| rs10048743 | 2 | 213890232 | T | G | 0.137 | -0.231 | 0.041 | 2.04E-08 | -0.035 | 0.023 | 0.126 |
| rs10200680 | 2 | 223961877 | T | C | 0.143 | -0.248 | 0.042 | 4.96E-09 | 0.011 | 0.023 | 0.619 |
| rs1078324 | 5 | 149202268 | A | C | 0.051 | -0.713 | 0.078 | 7.11E-20 | 0.065 | 0.036 | 0.075 |
| rs10912578 | 1 | 173251856 | G | A | 0.682 | -0.247 | 0.031 | 1.65E-15 | -0.006 | 0.017 | 0.743 |
| rs1143679 | 16 | 31276811 | A | G | 0.116 | 0.582 | 0.040 | 5.03E-48 | -0.045 | 0.025 | 0.076 |
| rs12094036 | 1 | 183558174 | C | T | 0.923 | -0.329 | 0.058 | 1.37E-08 | 0.020 | 0.030 | 0.500 |
| rs12524498 | 6 | 31444187 | T | G | 0.019 | -0.673 | 0.121 | 2.48E-08 | 0.102 | 0.060 | 0.088 |
| rs13136219 | 4 | 102743687 | T | C | 0.377 | -0.174 | 0.028 | 3.50E-10 | -0.014 | 0.016 | 0.394 |
| rs13332649 | 16 | 85966683 | G | A | 0.783 | -0.315 | 0.038 | 5.43E-17 | 0.026 | 0.019 | 0.175 |
| rs143123127 | 17 | 38007190 | A | G | 0.031 | 0.470 | 0.084 | 2.23E-08 | -0.071 | 0.047 | 0.127 |
| rs1464446 | 3 | 146601295 | T | G | 0.183 | -0.329 | 0.040 | 2.79E-16 | 0.029 | 0.020 | 0.154 |
| rs150180633 | 6 | 31010047 | T | C | 0.014 | 0.928 | 0.069 | 2.66E-41 | 0.045 | 0.079 | 0.571 |
| rs17849501 | 1 | 183542323 | T | C | 0.051 | 0.811 | 0.050 | 1.81E-59 | -0.001 | 0.039 | 0.987 |
| rs2431697 | 5 | 159879978 | C | T | 0.561 | -0.223 | 0.029 | 2.60E-14 | 0.031 | 0.016 | 0.049 |
| rs2459611 | 2 | 191939187 | T | C | 0.107 | 0.261 | 0.045 | 7.62E-09 | -0.051 | 0.025 | 0.043 |
| rs2573219 | 2 | 233288667 | C | A | 0.907 | 0.588 | 0.043 | 1.13E-42 | -0.022 | 0.030 | 0.461 |
| rs268124 | 2 | 65654364 | T | C | 0.269 | 0.186 | 0.032 | 8.60E-09 | -0.033 | 0.018 | 0.062 |
| rs28361029 | 6 | 31220203 | A | G | 0.070 | -0.386 | 0.061 | 3.27E-10 | -0.010 | 0.033 | 0.757 |
| rs34703115 | 2 | 40282854 | C | T | 0.968 | -0.616 | 0.105 | 4.08E-09 | 0.066 | 0.045 | 0.144 |
| rs35000415 | 7 | 128585616 | T | C | 0.114 | 0.588 | 0.042 | 1.86E-45 | -0.011 | 0.025 | 0.662 |
| rs35251378 | 19 | 10459969 | A | G | 0.291 | -0.236 | 0.032 | 3.61E-13 | -0.016 | 0.018 | 0.372 |
| rs353608 | 11 | 35101738 | G | A | 0.528 | 0.186 | 0.028 | 2.93E-11 | 0.005 | 0.016 | 0.743 |
| rs3747093 | 22 | 21984379 | A | G | 0.212 | 0.262 | 0.035 | 2.88E-14 | 0.005 | 0.020 | 0.811 |
| rs4388254 | 5 | 133428601 | T | C | 0.049 | 0.378 | 0.060 | 3.71E-10 | -0.010 | 0.038 | 0.785 |
| rs4661543 | 1 | 15229101 | G | T | 0.874 | 0.274 | 0.042 | 9.40E-11 | -0.031 | 0.024 | 0.194 |
| rs4916215 | 1 | 173314540 | T | C | 0.264 | 0.223 | 0.034 | 5.07E-11 | -0.018 | 0.018 | 0.315 |
| rs58688157 | 11 | 625085 | G | A | 0.738 | -0.223 | 0.034 | 2.97E-11 | -0.011 | 0.032 | 0.728 |
| rs58721818 | 6 | 138243739 | T | C | 0.031 | 0.658 | 0.076 | 3.38E-18 | -0.025 | 0.046 | 0.585 |
| rs6671847 | 1 | 161478810 | A | G | 0.480 | 0.199 | 0.029 | 6.64E-12 | 0.015 | 0.016 | 0.350 |
| rs6889239 | 5 | 150457771 | C | T | 0.747 | 0.278 | 0.032 | 2.19E-18 | -0.027 | 0.018 | 0.145 |
| rs7097397 | 10 | 50025396 | A | G | 0.379 | -0.186 | 0.029 | 8.60E-11 | 0.013 | 0.016 | 0.424 |
| rs73050535 | 12 | 5012503 | T | C | 0.022 | -0.713 | 0.124 | 9.11E-09 | 0.000 | 0.057 | 0.996 |
| rs73068668 | 19 | 55763262 | A | G | 0.076 | -0.315 | 0.057 | 4.40E-08 | 0.009 | 0.068 | 0.895 |
| rs7768653 | 6 | 106574794 | T | C | 0.398 | -0.207 | 0.030 | 3.11E-12 | 0.017 | 0.016 | 0.288 |
| rs7823055 | 8 | 55511676 | T | G | 0.424 | -0.351 | 0.029 | 1.64E-34 | -0.008 | 0.016 | 0.630 |
| rs7899626 | 10 | 63825561 | T | C | 0.318 | 0.182 | 0.033 | 4.19E-08 | 0.010 | 0.017 | 0.550 |
| rs9274357 | 6 | 32632457 | T | C | 0.200 | 0.457 | 0.035 | 1.28E-38 | 0.057 | 0.030 | 0.057 |
| rs9852014 | 3 | 129084581 | G | A | 0.926 | 0.621 | 0.049 | 2.26E-36 | -0.008 | 0.030 | 0.782 |

## Supplementary Figures


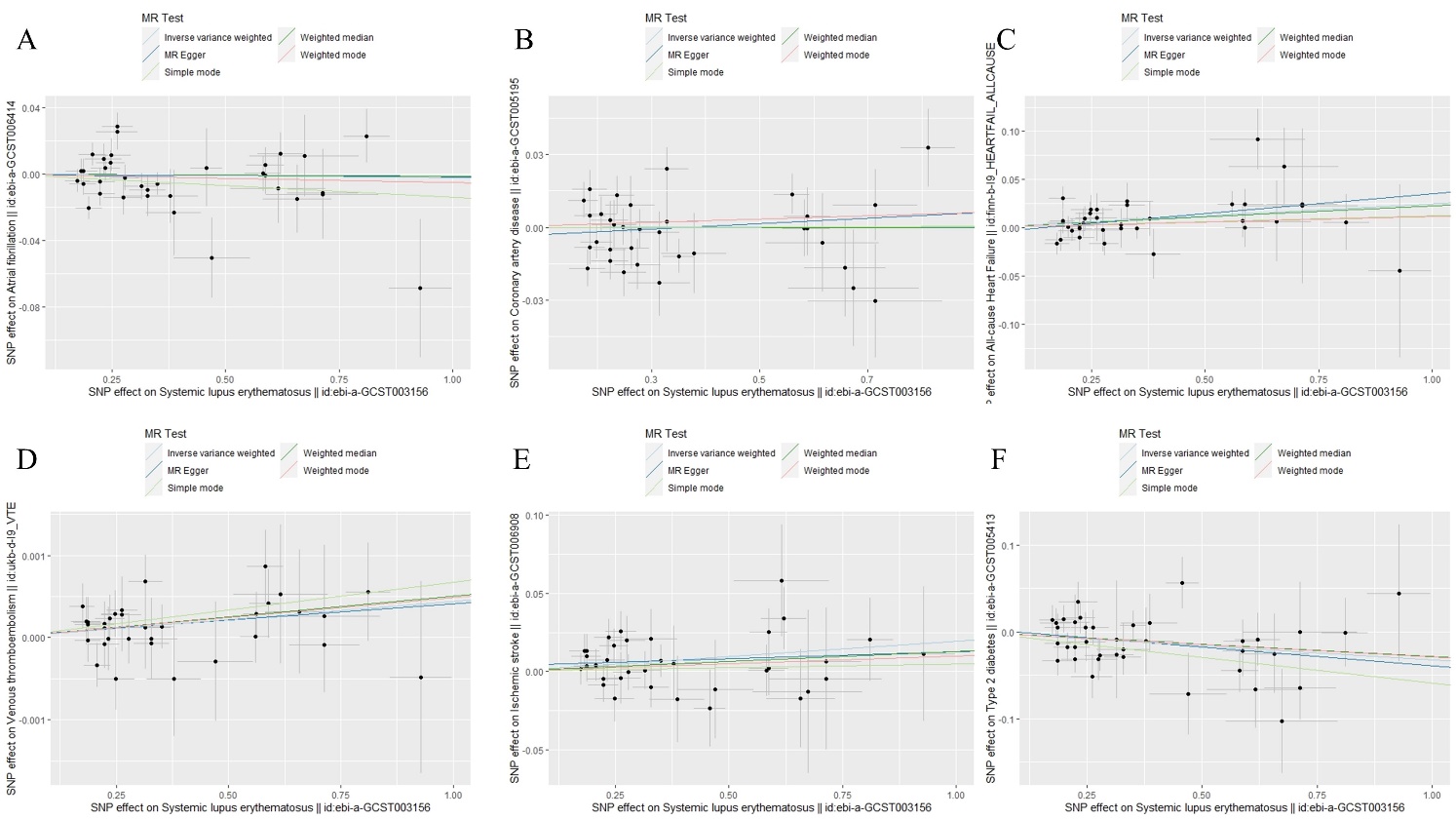


Supplementary Figure 1 Scatter plot: (A) SLE and AF; (B) SLE and CAD; (C) SLE and HF; (D) SLE and VTE; (E) SLE and IS; (F) SLE and T2DM


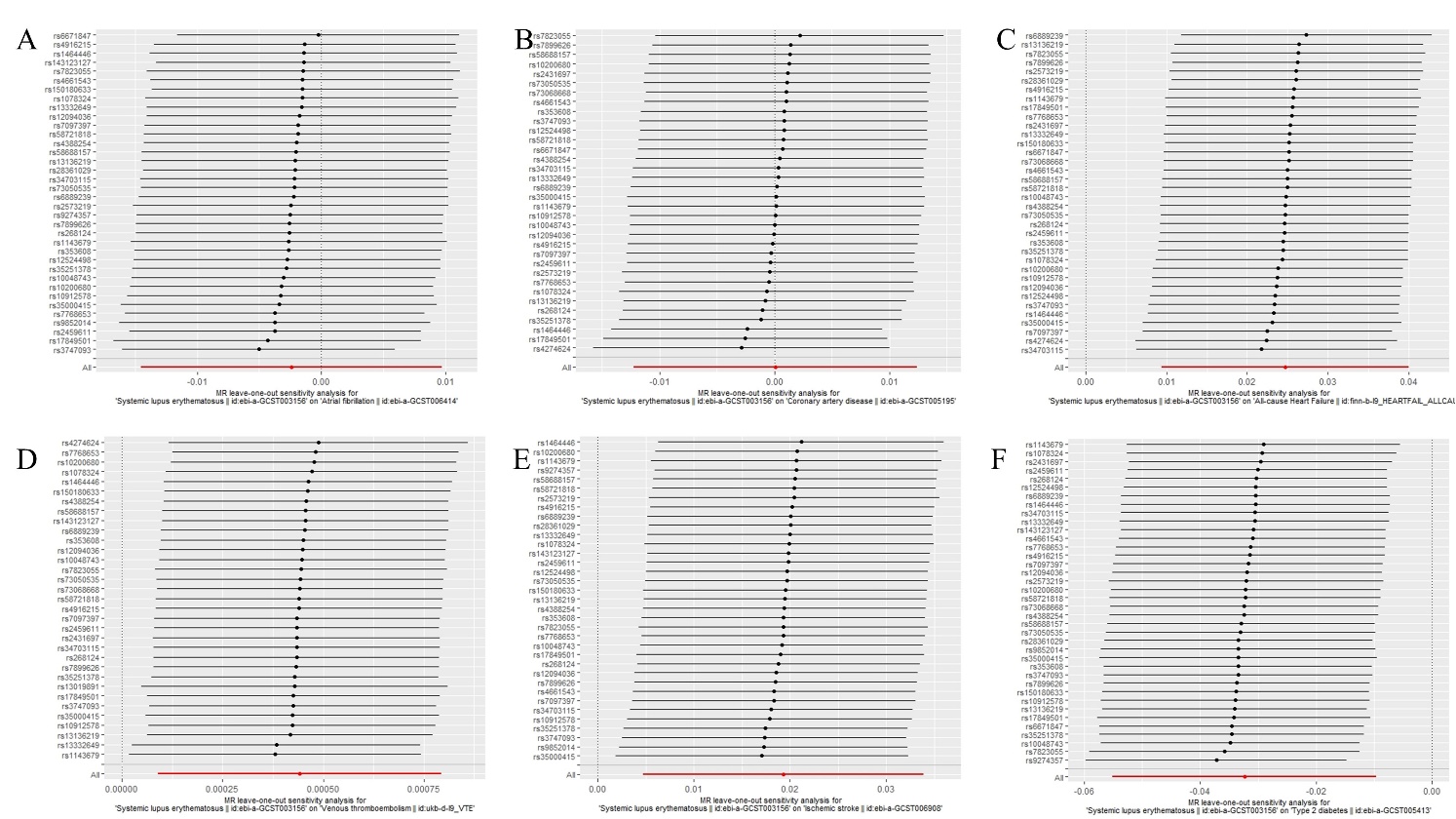


Supplementary Figure 2 Leave-one-out sensitivity analysis: (A) SLE and AF; (B) SLE and CAD; (C) SLE and HF; (D) SLE and VTE; (E) SLE and IS; (F) SLE and T2DM


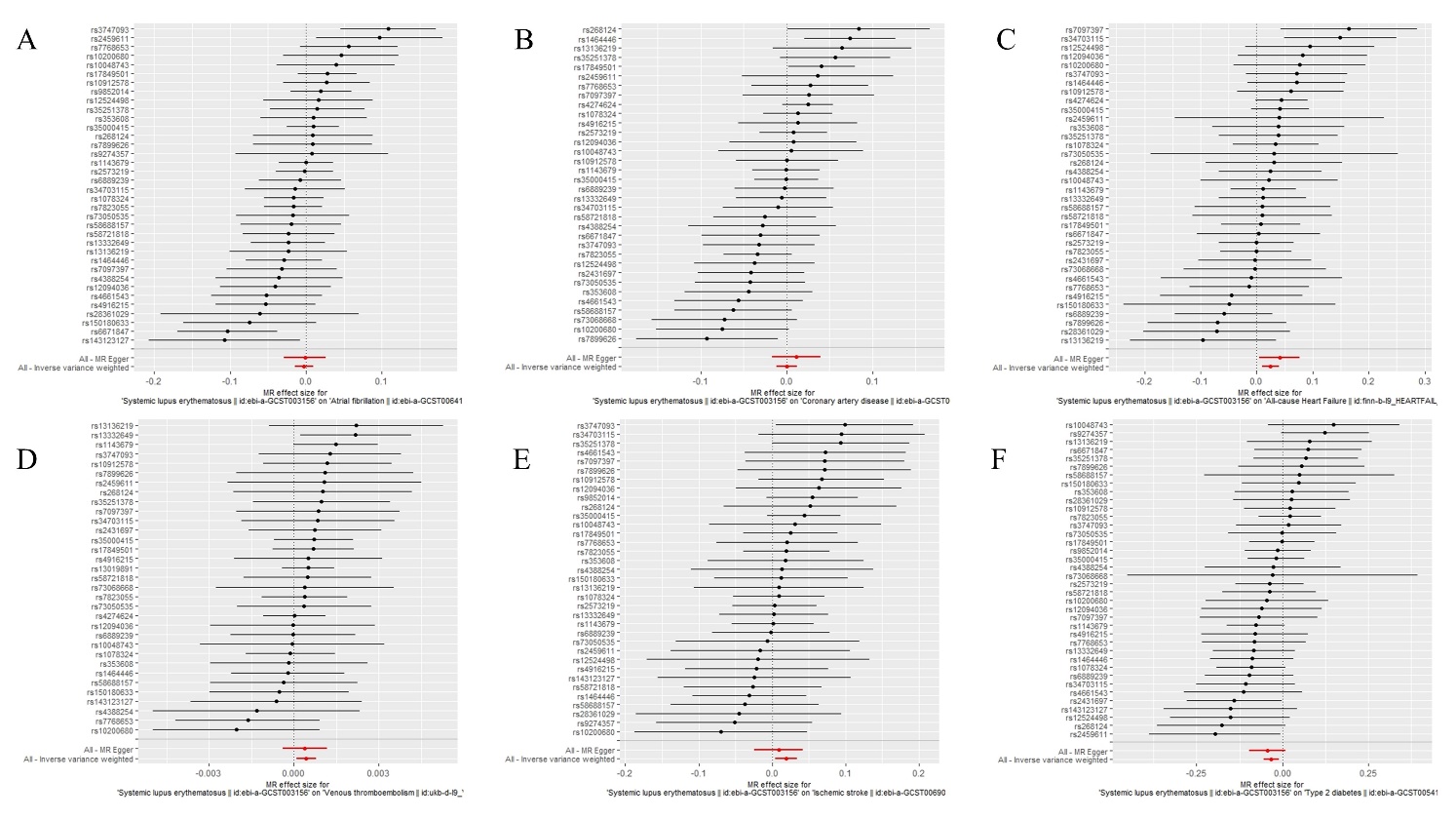


Supplementary Figure 3 Forest plot: (A) SLE and AF; (B) SLE and CAD; (C) SLE and HF; (D) SLE and VTE; (E) SLE and IS; (F) SLE and T2DM


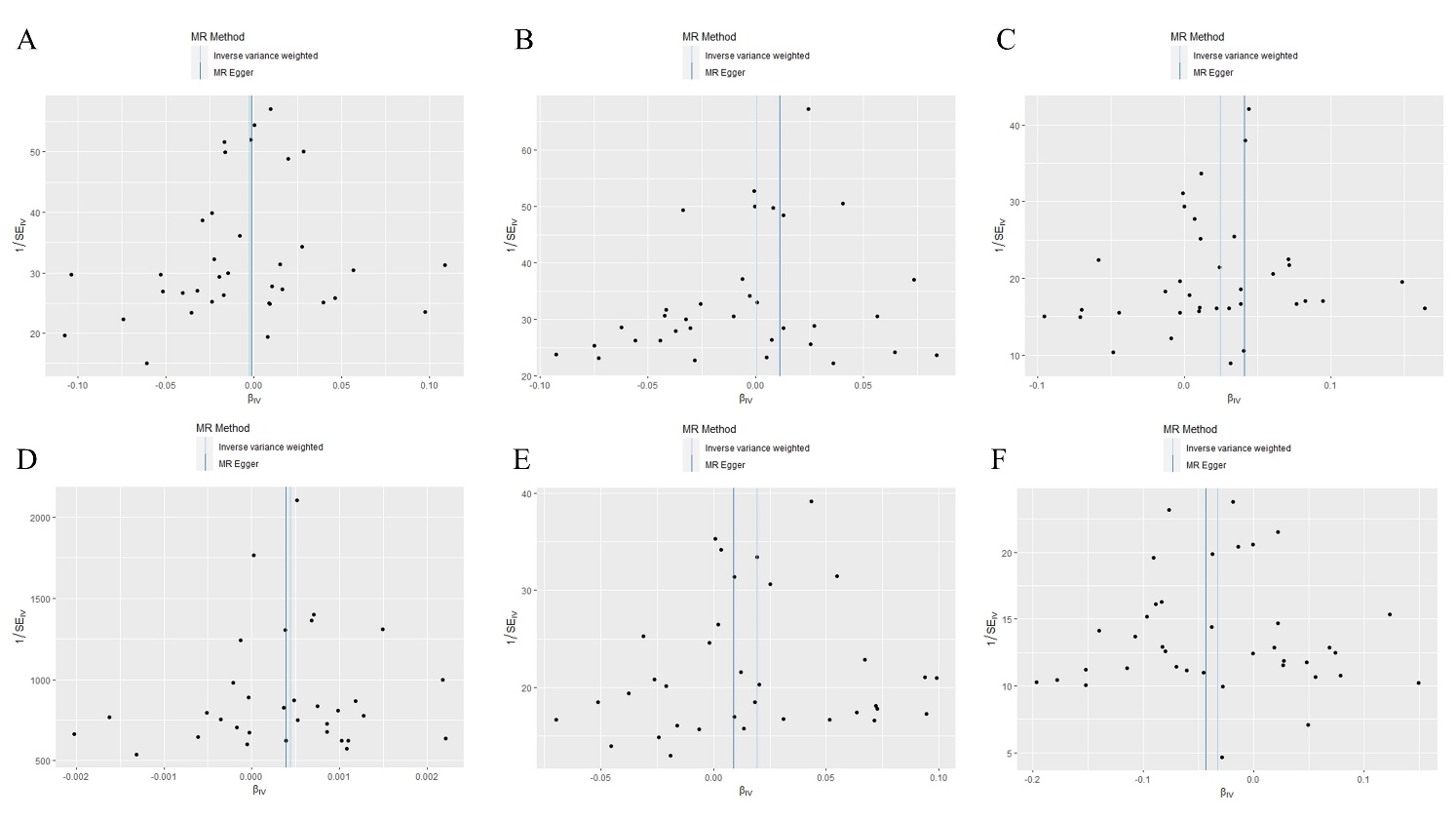


Supplementary Figure 4 Funnel plot: (A) SLE and AF; (B) SLE and CAD; (C) SLE and HF; (D) SLE and VTE; (E) SLE and IS; (F) SLE and T2DM
